# Exploring Associations between Stressors and Burnout in Trainee Doctors During the COVID-19 Pandemic in the UK

**DOI:** 10.1007/s40596-022-01660-x

**Published:** 2022-06-03

**Authors:** Anli Yue Zhou, Mark Hann, Maria Panagioti, Mumtaz Patel, Raymond Agius, Martie Van Tongeren, Aneez Esmail, Peter Bower

**Affiliations:** 1 University of Manchester, Manchester, UK; 2 Health Education England, Manchester, UK

**Keywords:** Covid-19, Burnout, Stressors, Trainee doctors, Training

## Abstract

**Objective:**

The authors examined associations between stressors and burnout in trainee doctors during the COVID-19 pandemic.

**Methods:**

An anonymous online questionnaire including 42 questions on general and pandemic-specific stressors, and the Maslach Burnout Inventory-Health Services Survey (MBI-HSS), was sent to 1000 randomly selected trainee doctors in North-West England. Main outcomes were burnout scores that were stratified into Emotional Exhaustion (EE), Depersonalisation (DP), and reduced Personal Accomplishment (PA) and associations between stressors and burnout using stepwise regression analysis.

**Results:**

A total of 362 complete responses were received giving a response rate of 37%. Mean scores for EE, DP, and PA derived from the MBI-HSS were 27.7, 9.8, and 34.3 respectively. Twenty-three stressors were found to be associated with burnout dimensions. “Increase in workload and hours due to COVID-19,” “Poor leadership and management in the National Health Service,” and “Not feeling valued” were found to have strong associations with burnout dimensions. Only “Not confident in own abilities” was found to be associated with all burnout dimensions.

**Conclusions:**

Associations with burnout were found to be identified in a range of work, pandemic, and non-work-related stressors, supporting the need for multi-level interventions to mitigate burnout.

Burnout is the consequence of long-term occupational stress, characterized by three subcomponents: Emotional Exhaustion (EE) (feeling of energy depletion or exhaustion), Depersonalisation (DP) (increased mental distance from one’s job, or feelings of negativism or cynicism related to one’s job), and perceived reduced Personal Accomplishment (PA) (reduced professional efficacy) [1].

Trainee doctors are qualified doctors who are engaged in postgraduate training such as interns and residents and are in the early stages of their medical careers. They have been found to experience a range of different stressors in their working lives [2, 3]. Furthermore, there is evidence to suggest doctors in the early stages of their career may be at a higher risk of burnout [4].

The COVID-19 pandemic has posed unprecedented challenges to healthcare systems worldwide and has not only exacerbated the pre-existing stressors impacting on doctors, but also introduced pandemic-related stressors [5]. Previous pandemic-related studies have focused on the prevalence of mental health in healthcare workers or specific specialties, and only a few studies have involved trainee doctors and which stressors affect them [6, 7]. The recent pandemic has highlighted the ongoing pressures experienced by trainee doctors in the United Kingdom (UK), with a third of trainee doctors reporting high levels of burnout due to work and nearly 60% reporting frequently feeling worn out at the end of the working day [8]. Hence, it is important to identify which stressors are associated with high levels of burnout in order to guide interventions to address burnout as the COVID-19 pandemic continues to put pressure on healthcare system.

Our study therefore aims to identify which stressors are relevant to burnout in trainee doctors during the pandemic.

## Methods

In the UK, all trainee doctors are employed in standardized training posts and follow set training pathways within a region to enable them to become accredited specialists within their chosen specialty. All trainee doctors undertake a 2-year foundation training rotation that provides experience in various specialties. Following this, trainee doctors join a specialty pathway that usually involves core training (2–3 years) followed by more specialized training (3–6 years). General practice trainees engage in a separate 3-year program after completing their foundation training, whereas specialty training can take more than 8 years.

A random sample of 1000 trainee doctors were selected from the eligible population of 7523 trainee doctors working in the North-West of England using STATA 14 [9].

A pre-warning email and participant information sheet were sent on 25/06/21 to inform 1000 randomly selected trainee doctor participants about the upcoming questionnaire. The formal invitation containing the online questionnaire link was sent to participants via email on 28/06/21. Non-respondents received 2 additional reminders by email. The survey was live between 28/06/21 and 03/08/21. All trainee doctors who completed the questionnaire were given a £20 shopping voucher for their participation.

The questionnaire consisted of 71 items covering 7 demographic questions (sex, age interval, specialty, grade, full-time status, ethnicity, and place of qualification), a list of general stressors as well as pandemic-related stressors experienced over the past month (42 questions using a 7-point Likert scale), and the Maslach Burnout Inventory-Health Services Survey (MBI-HSS) (22 questions) [10].

Methodology around developing the stressors questionnaire has been documented previously [11]. In brief, general stressors included 25 questions synthesized from previous research [2, 3] and a summary of the 25 questions has been previously published [11]. Five additional general stressors were added onto the questionnaire after receiving feedback from participants from a previous survey sent out in July 2020 and this included “ Financial remuneration does not reflect workload,” “Poor leadership and management in the National Health Service,” “Financial Worries,” “Workplace bullying and harassment/discrimination,” and “Negative impact of work on my health.” Twelve pandemic-related stressors that did not overlap with the general stressors were also included in the questionnaire. The methodology involved in creating these questions has been described in a previous publication [11]. Burnout was measured using the MBI-HSS, which is a validated 22-item questionnaire consisting of 3 subcomponents (EE, DP, and PA) and is the gold standard for measuring burnout [4, 10].

Backwards stepwise regression was undertaken to identify which stressors were associated with the burnout dimensions (EE, DP, and PA). General stressors and pandemic-related stressors were analyzed independently. Age interval, full-time status, gender, ethnicity, place of qualification, speciality, and grade were controlled for in the analysis. A forwards stepwise regression model confirmed stepwise regression results were consistent and did not identify any additional stressors. A two-sided *p*-value of ≤5% was considered statistically significant. Bootstrapping was undertaken to estimate model standard errors as it makes minimal assumptions about the distribution of the observed data.

Beta coefficients were also reported in order to determine the relative strength of association of each stressor with the burnout dimensions. For EE and DP, a stronger positive association is suggestive of a worse outcome whereas with PA, a stronger negative association is suggestive of a worse outcome. Data analysis was undertaken using STATA, version 14 (StataCorp) [9].

Approval was received from the University of Manchester Research Ethics Committee (UREC5-2016-0093-462) and the research committee at Health Education England North-West. Consent was implied by survey completion.

## Results

One thousand invitations were sent, out of which 9 invalid email addresses were identified. Four hundred and thirty-eight responses were received, out of which 31 were empty responses, 34 were incomplete responses, and 11 were duplicate responses. A total of 362 complete responses were collected out of 991 invitations during the study period giving a response rate of 37%.

Demographic data are presented in Table 1. Respondents were representative of the trainee doctor population in terms of gender, full-time status, and country of qualification. Some differences were identified between the respondents and the trainee doctors’ population in relation to speciality and grade (Table 1), and as a result, weights were derived in an attempt to make the respondent sample more reflective of the population that it is representing. However, weighted analyses did not result in different conclusions to the unweighted analyses and so only the latter are presented for simplicity.

**Table 1:** Demographic results

Demographics (*n*=362)	Frequency (%)	Comparison with the North-West trainee doctor population (*n*=7523)
Age		
≤30 years old	175 (48.3%)	Not available
31–35	121 (33.4%)	Not available
≥36	66 (18.2%)	Not available
Gender		
Female	191 (52.8%)	4041 (53.7%)
Male	169 (46.7%)	3402 (45.2%)
Unknown	2 (0.6%)	80 (1.1%)
Grade		
Foundation	57 (15.7%)	1692 (22.5%)
Core training year 1–2	90 (24.9%)	2312 (30.7%)
Specialty training/core training year 3	98 (27.1%)	1246 (16.6%)
Specialty training year 4/5	59 (16.3%)	1194 (15.9%)
Specialty training year 6+	58 (16.0%)	1076 (14.3%)
Unknown	-	3 (0.04%)
Specialty		
Anesthetics	27 (7.5%)	587 (7.8%)
Pathology	2 (0.6%)	87 (1.2%)
Foundation	16 (4.4%)	1692 (22.5%)
Other	18 (5.0%)	67 (0.9%)
Obstetrics and gynecology	18 (5.0%)	436 (5.8%)
Emergency medicine	23 (6.4%)	366 (4.9%)
General practice	99 (27.3%)	1014 (13.5%)
Medicine	61 (16.9%)	1412 (18.8%)
Surgery	30 (8.3%)	645 (8.6%)
Pediatrics	21 (5.8%)	574 (7.6%)
Psychiatry	26 (7.2%)	409 (5.4%)
Ophthalmology	4 (1.1%)	74 (1.0%)
Radiology	7 (1.9%)	160 (2.1%)
Full-time status		
Full time	299 (82.6%)	6420 (85.3%)
Part time	63 (17.4%)	1103 (14.7%)
Ethnicity^a^		
White	183 (50.6%)	Not available
Non-white	170 (47.0%)	Not available
Place of graduation^b^		
UK	278 (76.8%)	5538 (73.6%)
Non-UK	84 (23.2%)	1441 (19.2%)

^a^9 participants preferred not to disclose and therefore were not included

^b^7.2% (*n*=544) were categorized as unknown place of graduation

Mean scores for EE, DP, and PA were 27.7 (SD 11.5), 9.8 (SD 7.1), and 34.3 (SD 7) respectively. Median scores for EE, DP, and PA were 28 (range 4–54), 8 (range 0–30), and 35 (4–48) respectively with 52.3%, 40.1%, and 44.2% of the scores meeting the high burnout criteria for EE, DP, and PA respectively [4, 10].

Stressors that were significant at the 5% level (*p*<0.05) are presented in Table 2. Twenty-three stressors were found to be associated with burnout dimensions but only “Not confident in own abilities” was found to be associated with all 3 burnout dimensions. Strongest associations with EE, DP, and PA were “Increase in workload and hours due to COVID-19” (*β*=0.46), “Not confident in own abilities” (*β*=0.36), and “not feeling valued” (*β*=−0.24).

**Table 2:** Stressors that were significant at the 5% level using backwards stepwise regression modelling ^a^using bootstrapped percentile confidence intervals

General stressors	Emotional exhaustion		Depersonalisation		Reduced personal accomplishment	
	Coefficient (95% CI)^a^	Beta	Coefficient (95% CI)^a^	Beta	Coefficient (95% CI)^a^	Beta
Not confident in own abilities	1.23 (0.71, 1.74)	0.23	1.13 (0.65, 1.54)	0.36	−0.89 (−1.31, −0.51)	−0.19
Fatigue	1.05 (0.19, 1.79)	0.22	-	-	-	-
Poor worklife balance	1.52 (0.72, 2.19)	0.17	-	-	-	-
High workload	0.72 (−0.003, 1.42)	0.15	-	-	-	-
No control over work	1.08 (0.46, 1.74)	0.13	0.53 (0.091, 0.96)	0.13	-	-
Negative impact of work on my health	1.06 (0.40, 1.75)	0.12	-	-	-	-
No senior support	0.63 (0.03, 1.16)	0.12	-	-	-	-
Workplace bullying and harassment/discrimination	0.77 (0.18, 1.33)	0.05	-	-	-	-
Poor leadership and management in the National Health Service (NHS)	-	-	0.67 (0.27, 1.05)	0.22	-	-
Cannot take sick leave	-	-	0.49 (0.067, 1.04)	0.09	-	-
Cannot plan annual or study leave	-	-	0.36 (0.04, 0.74)	0.07	-	-
Work commute	-	-	0.38 (0.05, 0.71)	0.01	-	-
Fear of making mistakes	-	-	−0.42 (−0.85, −0.04)	−0.07	-	-
Not feeling valued	-	-	-	-	−0.94 (−1.46, −0.42)	−0.24
Concerns are not addressed	-	-	-	-	−0.69 (−1.12, −0.07)	−0.15
Career development and progression	-	-	-	-	0.53 (0.06, 1.06)	0.09
COVID-19-related stressors	Emotional exhaustion		Depersonalisation		Reduced personal accomplishment	
	Coefficient (95% CI)^a^	Beta	Coefficient (95% CI)^a^	Beta	Coefficient (95% CI)^a^	Beta
Increase in workload and hours due to COVID-19	2.72 (1.99, 3.36)	0.46	-	-	-	-
Training disrupted by COVID-19	1.02 (0.42, 1.62)	0.17	-	-	-	-
Feeling isolated due to COVID-19	0.68 (−0.03, 1.28)	0.13	0.59 (0.21, 0.97)	0.16	-	-
Working beyond normal scope due to COVID-19	-	-	0.86 (0.41, 1.31)	0.21	−0.92 (−1.39, −0.44)	−0.19
Uncertainty around COVID-19 information	-	-	-	-	−0.80 (−1.24, −0.31)	−0.21
Personal protective equipment (PPE) concerns	-	-	-	-	0.47 (0.05, 0.88)	0.09
Concerns about non-COVID-19 patient care	-	-	-	-	0.70 (0.08, 1.31)	0.17

## Discussion

This cross-sectional study has identified that a range of different stressors were found to be associated with burnout (especially EE) in trainee doctors. Work-related stressors that emerged during the pandemic were found to have stronger associations with burnout when compared to non-work-related pandemic stressors.

“Increase in workload and hours due to COVID-19” was found to have the strongest association with burnout (EE) which is consistent with the unprecedented pressures on healthcare systems due to the COVID-19 pandemic [5]. This could also be related to trainee doctors having to juggle training commitments alongside their day-to-day clinical demands, which are both factors that have been found to be associated with burnout in trainee doctors previously [2]. Our study also identified “Training disrupted by COVID-19” to be associated with EE which suggests the negative impact of COVID-19 on training remains despite contingency plans such as flexibility with annual appraisal and creation of virtual learning environment to ease training pressures [8]. A recent survey showed that nearly 50% of trainee doctors report ongoing difficulties with missed training opportunities during the pandemic [8], suggesting unaddressed training concerns can heighten the impact of burnout. These findings also suggest the need to develop proactive robust contingency plans for crisis management.

We found that trainee doctors who were concerned about making mistakes were less likely to experience DP and this finding seems to contrast with previous research on patient safety incidents and burnout [12]. However, these studies have focused on measuring subsequent burnout after the incident had occurred [12] whereas in our study, we assessed the concerns around potentially making mistakes and did not objectively measure the number of patient safety incidents. Furthermore, the association found in the study was small (*β*=−0.07), and therefore, its clinical significance is unclear at present. To build on this finding, further research is required to establish the relationship between fear of making mistakes, burnout, and actual patient safety incidents. Another stressor associated with DP was “Poor leadership and management in the NHS” which is consistent with the existing literature that has emphasized the importance of good physician leadership to prevent burnout in doctors [13]. However, mechanisms through which non-physician leadership contributes to burnout in the UK are unclear and may merit further research.

Stressors found to be associated with burnout in our study were mostly associated with one subcomponent of burnout but “Not confident in own ability” was associated with all subcomponents of burnout which is consistent with previous research [2], suggesting those lacking in confidence and self-efficacy may be at a higher risk of burnout. Lack of confidence could impact on clinical decision-making and how they perceive clinical uncertainty in the workplace, which has also found to be a driver for burnout especially in more junior staff [14]. One area of support could include job crafting, which involves making changes to align the work with worker’s own abilities, and has been found to improve self-efficacy [15]. Job crafting interventions in healthcare workers can vary between studies and post-intervention assessments are measured only in the short term [16, 17], therefore making it difficult to assess generalizability of the intervention and the longer term benefits. Further research is therefore required to assess the format and feasibility of job crafting interventions for trainee doctors and its long-term benefits.

This study has identified a range of general and pandemic-specific stressors experienced by UK trainee doctors working and has explored the association of these stressors with burnout. Previous studies have focused on burnout in healthcare workers during the covid-19 pandemic [6, 7]; however, this is one of the very few studies that have focused specifically on doctors in training [18, 19]. While exploration of stressors in trainee doctors has been limited [18, 19], our study has included a comprehensive range of stressors identified in UK trainee doctors and the previous literature [2, 3]. This study therefore provides important findings, which can guide intervention development to mitigate burnout in trainee doctors.

This was a cross-sectional study, and therefore, causation cannot be assessed. The overall response rate was 37%, which is reflective of previous online surveys involving doctors [18, 20]. Although we attempted to improve response rates through adopting various methods such as a covering letter, advanced warning email, guaranteed incentive, and follow-up reminders, our response rates remained similar to other studies [18, 20]. Considering that trainee doctors are reporting high levels of burnout, survey fatigue could also have contributed to the response rate. Response bias may also have been present within our study as foundation doctors were found to be under-represented compared to other grades and specialties, and general practice trainees were found to be over-represented. Multiple factors may influence response bias and it may not be possible to mitigate all factors; however, when weighting was applied according to specialty and grade, the conclusions did not change when using a weighted analysis. Another limitation is that this study might have applied multiple hypothesis testing due to the nature of the Maslach Burnout Inventory, which does not sum scores and gives equal importance to all 3 subcomponents. Furthermore, there may have been potential interactions between the stressors and the demographic covariates. However, fitting interactions and numerous main effects in the regression analysis with the current sample size may have overloaded the regression model and future studies with larger sample sizes may be needed. Our study focused on trainee doctors only, but the COVID-19 pandemic has put unprecedented pressures on all medical professionals. Therefore, a potential area for further research could compare whether there may be differences in the types of stressors experienced between junior and senior doctors and which could provide additional insights into intervention design.

Our findings provide guidance on trying to address specific work-related and non-work-related stressors/components including proactive crisis management planning, job crafting, managing clinical uncertainty, and the role of leadership, and further support the evidence that multi-level interventions should be considered to mitigate the risk of burnout.
